# Know Your Heritage: Exploring the Effects of Fit in Cultural Knowledge on Chinese Canadians’ Heritage Identification

**DOI:** 10.3389/fpsyg.2018.02100

**Published:** 2018-11-05

**Authors:** Rui Zhang, Kimberly A. Noels, Richard N. Lalonde

**Affiliations:** ^1^Department of Psychology, Dickinson College, Carlisle, PA, United States; ^2^Department of Psychology, University of Alberta, Edmonton, AB, Canada; ^3^Department of Psychology, York University, Toronto, ON, Canada

**Keywords:** cultural fit, fit in cultural knowledge, enculturation, heritage identity, cultural values, the model minority stereotype, Chinese Canadians

## Abstract

In the present research, we introduce the notion of fit in cultural knowledge (FICK) – which we define as a match between the self and others in representing a cultural tradition. For ethnic minorities, FICK can be manifested in different degrees of matching their personal beliefs about their heritage culture with outgroup as well as ingroup beliefs about their heritage culture. We conducted two studies with the objective of exploring the potentially negative effects of FICK on Chinese Canadians’ heritage identification. In both studies, Chinese Canadian university students (*N* = 102; *N* = 156) indicated their personal beliefs about what values are normative in Chinese culture. Ingroup beliefs were assessed by beliefs about Chinese values that Chinese Canadians ascribed to their parents (Study 2), whereas outgroup beliefs were assessed by beliefs about Chinese values that were held by Euro-Canadians (Study 1) or that Chinese Canadians ascribed to Euro-Canadians (Study 2). The main findings based on a series of path models are as follows: (1) a stronger FICK generally predicted lower Chinese identification (centrality, ingroup ties, and affect), yet those negative effects were largely manifested in the openness to change versus conservation rather than in the self-transcendence versus self-enhancement value dimension. (2) The negative effects could be explained by Chinese Canadians’ experience of bicultural conflict (Study 1) and the frustration of continuity, meaning, and belonging identity motives (Study 2), suggesting that it matters which specific views of Chinese culture are matched in FICK. 3) Individuals who agreed with the perceived outgroup beliefs, and parental beliefs to a lesser extent, were more likely to apply the model minority stereotype to other Chinese Canadians (Study 2). Taken together, those findings demonstrate the challenges FICK presents to heritage identity maintenance among Chinese Canadian young adults. Implications for enculturation and cultural fit are discussed.

## Introduction

The dual concern about cultural change and maintenance becomes salient when people come into continuous contact with a culture other than their own. For immigrants and their offspring, cultural change is the process of adopting or acquiring the common practices and values of the settlement culture, while cultural maintenance entails continuing practices with the heritage culture. The transmission of heritage culture within immigrant families, which tends to occur in the settlement society for extended periods of time, is typically referred to as enculturation (Birman and Addae, 2015). Although a substantial amount of work shows that the majority of immigrants and their children tend to maintain their heritage culture (as well as participate in the settlement culture; Berry et al., 2006), it leaves open questions regarding the continuous process of enculturation, especially in relation to the learning of heritage traditions and the development of a heritage ethnic identity. How do young adults from immigrant families arrive at an understanding of their heritage culture? And how do their views of heritage culture relate to their heritage identity?

We attempt to understand Chinese Canadians’ personal beliefs about the normative values of Chinese culture and unpack their implications for Chinese ethnic identity. We first distinguish two sources that may inform Chinese Canadians’ personal beliefs about Chinese culture: beliefs about Chinese culture which are shared within the Chinese community (the ingroup source) and common beliefs about Chinese culture which are held by Canadians (the outgroup source). We then extend the perspective of cultural fit to introduce the notion of matching beliefs about the normative values of Chinese culture. That is, Chinese Canadians should vary in the extent which they incorporate those source beliefs into their personal views about Chinese culture. Importantly, we examine whether fit conceptualized as such affects Chinese Canadians’ identification with Chinese culture. In the studies reported below, we focus specifically on the fit between personal beliefs and perceived parental beliefs and fit between personal beliefs and actual or perceived outgroup beliefs regarding the normative values of Chinese culture. The question is what are the overall effects of fit in beliefs about Chinese cultural values on Chinese Canadians’ heritage identification?

### Cultural Fit and FICK

From the cultural psychological perspective, psychological tendencies and behaviors are attuned to sociocultural environments (Shweder, 1991; Markus and Kitayama, 2010). Although fit between personal and particular environmental characteristics (e.g., small groups and organizations) has been examined for some time (e.g., Kristof-Brown et al., 2005), cultural fit highlights a personal match with the broad sociocultural context. Cultural fit has been examined across diverse psychological constructs that are posited to vary across cultures, including personality (Eap et al., 2008; Fulmer et al., 2010), self-esteem (Heine and Lehman, 2004), emotions (De Leersnyder et al., 2011), and identities (Noels et al., 2010). Implicit in this notion is not merely the claim that different sociocultural contexts afford culture-specific ways of thinking, feeling, and acting, but that the fit itself is a contributor of physical and psychological well-being (Kitayama and Park, 2007) and that misfit precipitates malaise (Caldwell-Harris and Ayçiçegi, 2006; Dressler et al., 2018). The latter point has received much support in research in which cultural fit was assessed directly in terms of a match between individual tendencies and cultural norms (Fulmer et al., 2010; Stavrova, 2015; Bleidorn et al., 2016). A case in point is the work on emotional fit with culture, which shows that people can benefit from sharing a similar pattern of emotional experiences with others in their culture. Specifically, cultural fit of emotion has been associated with more positive relationships (De Leersnyder et al., 2014) and higher psychological well-being in domains most relevant to the realization of cultural goals such as autonomy and relatedness (De Leersnyder et al., 2015).

Extending the notion of cultural fit, we examine fit at the level of cultural knowledge, which we term fit in cultural knowledge (FICK). FICK differs from generic cultural fit in that it refers to consensus between the self and others on *knowledge* about a culture, rather than an alignment of one’s specific ways of thinking, feeling, or acting with those of general or close others. The distinction between FICK and the typical sense of cultural fit corresponds with cultural competence and cultural consonance in the cognitive theory of culture (Dressler, 2018; Dressler et al., 2018). Both cultural competence and cultural consonance acknowledge variation in the relation between an individual and their culture, yet with distinct points of emphasis. Cultural competence refers to variation in personal agreement with the cultural knowledge distributed within a group; a person may represent his or her culture similarly to or differently from how most others relate to the same culture. In contrast, cultural consonance emphasizes variation in behavioral enactment of the cultural knowledge or how the individual “puts that knowledge into practice” in his or her own life (Dressler et al., 2018, p. 10). Similar to cultural competence, we use FICK to denote the extent to which Chinese Canadians share their understanding of Chinese culture with ingroup and outgroup members. In the research presented here, we focus on FICK in cultural values, that is, how one’s personal belief matches other people’s beliefs about the normative values of Chinese culture.

**FIGURE 1:**
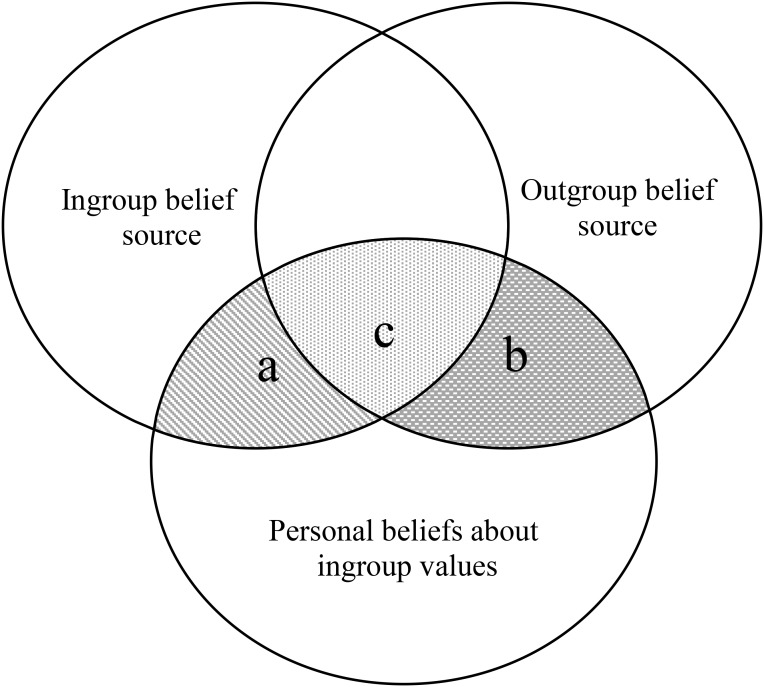
Visual representation of FICK: relations among personal beliefs and the ingroup and outgroup source beliefs.

We think FICK is a particularly useful concept in the context of enculturation because unlike young adults in their countries of origin, ethnic minorities are typically not formally socialized into their heritage culture, but rely heavily on their family and the local community. As such, FICK may take the form of internalizing his or her parents’ view of heritage culture. While socialization within immigrant families is a well-researched topic (Costigan et al., 2017), comparatively little is known of what view ethnic minorities acquire from their parents in relation to heritage culture, let alone the effects of matching personal beliefs with parental views. Meanwhile, what influences their personal beliefs extends beyond their own family or the local community. Ethnic minorities are also exposed to common beliefs about their ingroup held by the larger society, particularly those by the majority group. Thus, learning about their heritage could result in matching personal beliefs, to varying degrees, with both source beliefs. The relations among those three beliefs are visualized in Figure 1, with each belief represented by a circle. Most pertinent are the three shaded areas where the circles intersect. Areas marked as a and c correspond to the match between personal beliefs about the ingroup Chinese values and ingroup beliefs about them (ingroup FICK), while areas marked by b and c delineate the match between personal beliefs about the ingroup Chinese values and outgroup beliefs about them (outgroup FICK). The common area c is meant to represent the possibility that ingroup and outgroup beliefs overlap to some degree, which should cause the ingroup and outgroup FICK to be somewhat correlated as well.

Admittedly, the source beliefs depicted are overly simplified. The ingroup–outgroup distinction is not meant to suggest each source belief is internally homogenous or monolithic. For example, ingroup influence can vary along multiple dimensions such as socialization agent (vertical versus horizontal transmission, Phinney et al., 2001) and locality (local and direct exposure versus remote and indirect exposure, Ferguson et al., 2016); what is transmitted about heritage culture need not be in full agreement. Similarly, the outgroup source may refer to the views of the majority group or those of other ethnic minority groups. Nonetheless, we think such a rough distinction between ingroup and outgroup influence is helpful in drawing attention to the sociocultural context in which enculturation takes place. When it comes to making sense of one’s ethnic or racial identity, the cultural psychological perspective distinguishes an insider from an outsider view (e.g., Markus, 2010). While both views can be the basis of ideas and meanings that informs someone’s understanding of their heritage culture, the former refers to the shared understanding within the ingroup (e.g., ethnic peers or parents) and the latter amounts to imposed definitions by outsiders (typically the dominant group). For Chinese descendants living in the United States and Canada, enculturation consists of coming to terms with how Chinese or Chinese Americans/Canadians are viewed not only within their own communities, but also in the larger society. Thus, a unique challenge is to confront stereotypical images of being Chinese or Asian that are propagated and consumed in the public sphere (for research on media representation of Asian Americans, see Mok, 1998; Aoki and Mio, 2009).

As briefly summarized above, the effects of cultural fit have been largely positive. However, a few studies also reported negative findings (e.g., Ward et al., 2004; Fung et al., 2016). Those exceptions call into question whether cultural fit is optimal regardless of context and domain of matching. In the present research, we examine whether personal agreement with ingroup and outgroup members regarding the normative values of Chinese culture (i.e., FICK in cultural values) may interfere with Chinese Canadians’ ability to connect positively with Chinese culture. In particular, in terms of representing the ingroup source of FICK, given the extensive research on family socialization, we focus on the perceived parental view. For the outgroup source of FICK, we focus on the mainstream Canadian view held by European Canadians or perceived by Chinese Canadians.

### Heritage Ethnic Identity and Its Development

As the fundamental goal of enculturation is to prepare a person to be a competent and valuable member of a cultural community, it encompasses not only the acquisition of cultural knowledge and skills, but also the development of an identity rooted in the heritage culture. Ethnic identity refers to the subjective sense of belonging to an ethnic group (Phinney, 1990; Clément and Noels, 1992). Whereas identity is understood to be a key developmental task for adolescents and emerging adults (Arnett, 2000), there are unique challenges facing youth from ethnic minority backgrounds because they must grapple with identity issues related to their heritage culture and how their heritage ethnic identity fits with other socio-cultural group identities such as the mainstream cultural identity. We focus on one’s overall or global sense of identity with the heritage ethnic group. Much of the existing work has examined direct and indirect benefits of a strong heritage ethnic identity, including increased self-esteem and subjective well-being as well as reduced depression (Crocker et al., 1994; Umaña-Taylor et al., 2009; Bombay et al., 2010); it also protects ethnic minorities against negative psychological consequences of adverse experiences such as daily stress and discrimination (Shelton et al., 2005; Kiang et al., 2006; Bombay et al., 2010; Torres and Ong, 2010).

Given the generally salubrious effects of heritage ethnic identity, research has also examined contextual factors that facilitate or inhibit the development of a well-anchored heritage ethnic identity. One well-studied contextual factor that supports the development of heritage identity is family socialization (Hughes et al., 2006). One specific aspect is parental practices that are geared explicitly toward promoting cultural customs and traditions and ethnic pride. Those explicitly cultural socialization efforts have been associated with stronger heritage identity commitment (Phinney et al., 2001; Umaña-Taylor et al., 2006) in addition to other positive developmental outcomes. Relatedly, family socialization also creates the space (e.g., family gatherings and ethnic community) and tools (e.g., heritage language) that afford ethnic minority youth the opportunity to explore the meaning of their heritage ethnicity, a developmental stage important to establishing a well-grounded heritage ethnic identity (Phinney, 2006). Research also shows that parents transmit to children not only their personal values but also values that are perceived to be culturally important (e.g., Tam et al., 2012). Through ethnic socialization practices, parents of ethnic minorities likely also pass values that are considered important to their heritage culture. Thus, parental beliefs about heritage culture may constitute an ingroup source of cultural learning that informs ethnic minorities of what is more or less valued in their heritage culture. Given the many ways in which parents play in heritage socialization, seeing eye to eye with them in terms of what is valued in the heritage culture can be understood to serve positive ends, such as maintaining a shared reality (Hardin and Higgins, 1996; Wan et al., 2010), reducing intergenerational friction (Phinney et al., 2000), and increasing perceived cultural continuity over time (Sani et al., 2007). On the other hand, the perspective on heritage culture passed along by one’s parents may also be particularly antiquated (Kim et al., 1999), making it difficult for young adults living in a drastically different context from their country of origin to relate to their heritage. Moreover, wholeheartedly adopting the parental view may reflect a non-reflective stance toward the heritage culture, which could paradoxically undermine the ability to derive personal meaning. Given competing psychological forces, it is important to explore the overall effects of parental FICK on heritage identification.

Also pertinent to ethnic minorities’ understanding of their heritage culture is the potential role of outgroup beliefs. Outgroup beliefs may contain, to a great extent, common stereotypes associated with one’s heritage group, which have been shown to be one contextual factor that can inhibit the achievement of a positive sense of heritage ethnic identity. Growing up in a multiethnic society often means having to contend with stereotypical attributes associated with one’s group (Way et al., 2013). To the extent that those stereotypical attributes threaten a positive sense of self, routine encounters with them may evoke psychological distancing from one’s ethnic identity (Arndt et al., 2002; Yip, 2016). While the perils of internalizing negative stereotypes are understandable, it is less clear whether exposure to positive stereotypes would similarly increase disidentification. The latter question is particularly relevant to Asian Americans/Canadians due to the model minority stereotype, which seems to tout the success story of Asian Americans (Taylor and Stern, 1997; Paek and Shah, 2003). As previous research has documented some negative consequences of receiving positive stereotypes among Asian Americans (Cheryan and Bodenhausen, 2000; Siy and Cheryan, 2013) and given one study that found the influence of daily experience of stereotypes (regardless of valence) on heritage disidentification (Yip, 2016), we explore the potentially negative effects of outgroup FICK on heritage identification. In particular, our choice of cultural values to assess FICK makes it unlikely that outgroup appraisal of Chinese culture was unequivocally negative (see details below).

### The Present Research

The present research attempted to explore the potentially negative effects of FICK on heritage identification. Building on theorizing that maintains whether people become identified with a culture depends on how they evaluate the cultural content (values, beliefs, norms and etc.; Hong et al., 2007), we aimed to show what ethnic minorities know about the values of their heritage culture would predict how they subjectively position themselves in relation to that culture. Specifically, we focused on fit between personal beliefs and perceived parental beliefs (parental FICK, Study 2) and fit between personal beliefs and outgroup beliefs (outgroup FICK, Studies 1 and 2). Given the exploratory nature of this research, we also tested theoretically plausible mechanisms by which FICK may lead to lower heritage identification. In Study 1, we investigated the possibility that outgroup FICK reflects specific ways of viewing Chinese culture; agreeing with those ways of viewing Chinese culture leads Chinese Canadians to experience bicultural identity conflict that stems from perceived cultural incompatibility, which lowers heritage identification. In Study 2, we adopted a theoretical perspective that seeks to understand why people are drawn toward some identities, but away from others. To the extent that FICK fails to satisfy some motives that are known to energize identity construction, ethnic minorities may not feel motivated to embrace their heritage culture. We empirically derived those identity motives that may be thwarted by FICK. Finally, with regard to the study population, we focused on emerging adults in universities, ranging mostly from 17 to about 22 years of age. For ethnic minorities, the need to extensively explore the implications of their ethnic identity could well continue far beyond adolescence and into early adulthood (Phinney, 2006). Our population of interest was Chinese Canadians, who represent the most common non-European ethnic ancestry in Canada (Statistics Canada, 2016; also see Costigan et al., 2009).

In both studies, we drew on Schwartz’s (1992) model of values in order to assess beliefs about normative Chinese values. Values are defined as desirable, *trans*-situational goals that serve as guiding principles in people’s lives (Schwartz, 1992). Because values refer to generally desirable end-states, this means that beliefs about a culture assessed via values are largely positive as well. Schwartz postulated and validated 10 motivationally distinct types of values that form a circumplex model of motivational oppositions and compatibilities (Schwartz, 1992). Those 10 value types can be organized in terms of two higher-order bipolar dimensions: openness to change versus conservation and self-transcendence versus self-enhancement. The first dimension opposes autonomy of thought and action (value types of self-direction and stimulation) with self-restraint and maintaining the status quo (value types of tradition, security, and conformity), while the second dimension contrasts the pursuit of group interests (value types of benevolence and universalism) with the advancement of personal goals (value types of achievement and power; see Schwartz, 1994). Although Schwartz’s value survey was initially developed to assess values held by individuals, it has been successfully adapted to measure people’s beliefs about culturally normative values (Wan et al., 2007). Thus, the use of Schwartz’s values allowed us to consider how Chinese culture is understood on those two bipolar dimensions. Accordingly, we operationalized FICK as the degree of similarity in openness to change versus conservation and self-transcendence versus self-enhancement separately. Previous research has generally found Chinese and Canadian cultures to differ or at least perceived to differ on both dimensions (Schwartz, 2008; Fung et al., 2016). Separating those two dimensions allows us to test how the broadly construed value dimensions would play a role in linking FICK with heritage identification.

Finally, like other group identities, ethnic identity is a multidimensional construct (Ashmore et al., 2004). There have been a number of content-focused models that proposed dimensions comprising the content of ethnic identity (Schwartz et al., 2014). We adopted a tripartite model that has been useful for conceptualizing components common to many group identities including ethnic identity (Cameron, 2004). According to this model, ethnic identity consists of three interrelated sources: centrality (membership importance), affect (positive evaluation of the group), and ties (strength of connection to group members). In the present research, we empirically assessed heritage identification with those three components.

## Study 1: Outgroup Fick

As an initial test of the potentially negative effects of FICK, we operationalized FICK as the match between a Chinese Canadian’s personal belief about the ingroup Chinese values and common Canadian beliefs (outgroup FICK). While personal beliefs came directly from a sample of Chinese Canadians, outgroup beliefs were elicited from a sample of European Canadians. Central to our goal was the mapping out of possible mechanisms by which FICK could lead to lower heritage identification. We tested a mediation model in which the negative effects of outgroup FICK on heritage identification were mediated by the experience of bicultural identity conflict. Our working assumption was that outgroup FICK implies particular ways of viewing Chinese culture. For instance, to the extent that other Canadians view Chinese culture as higher on conservation (vs. openness to change), a close match for a Chinese Canadian person on this dimension would indicate personal agreement with such a characterization. Having this personal belief might then put the contrast between Chinese and Canadian cultures in sharper relief, which creates the perception of a large cultural divide and difficulty in integrating cultural identities (Huynh et al., 2011). Decreasing identification with or even disidentification from a group represents a cognitively facile strategy of managing identity conflict as it reduces the self-relevance of one identity within a bicultural (Yampolsky et al., 2016). In other words, a stronger outgroup FICK may lead to lower identification with Chinese culture because it creates bicultural identity conflict that stems from perceived incompatibility of cultural values.

The above reasoning also suggests that outgroup FICK is most likely to predict identity conflict on dimensions where Chinese and Canadian cultures differ or are perceived to differ widely. Although previous research points to differences or perceived differences on both dimensions (Schwartz, 2008; Fung et al., 2016)^1^, it is an empirical question whether identity conflict would be affected by perceived differences on both equally. Thus, we relied on exploratory analyses to see whether outgroup FICK on both dimensions would be associated with identity conflict. Finally, previous research failed to find any negative association between identity conflict and heritage cultural identification (Benet-Martínez and Haritatos, 2005; Cheng et al., 2006). Because the past research assessed cultural identities primarily in terms of centrality, we examined associations between identity conflict and all three components of heritage identity.

### Method

#### Participants^2^

The sample consisted of 102 Chinese Canadians (58.4% women; one did not indicate gender) and 49 European Canadians (67.3% women) recruited from introductory psychology classes at a western Canadian university. The mean age was 18.91 years (*SD* = 1.65). All European Canadians and half of the Chinese Canadians were born in Canada. Within the Chinese Canadian sample, all but three reported having at least one foreign-born parent (one did not indicate).

#### Measures

Participants completed a survey, which included the following instruments and some demographic questions, in group-testing sessions.

##### Cultural values

All participants completed the SVS, which provided them with a list of 58 values that assess the two bipolar dimensions (Schwartz, 2009). Unlike its typical use for assessing personal values, participants were explicitly asked to rate their perceptions of Chinese culture with different prompts for the two groups. The Chinese Canadians were instructed to rate the values for a typical Chinese person holding Chinese values. Because we were interested in their spontaneous representations of Chinese culture, we did not specify whether this person was Chinese or Chinese Canadian. The European Canadians were asked to focus on how a typical Chinese person holding Chinese values is viewed in the Canadian society and then rate each SVS value for that target Chinese person. All responses were provided on an 8-point scale, ranging from 0 (*not important*) to 7 (*extremely important*). The original SVS also had the option of selecting -1 to indicate values opposed to one’s principles. We did not include that rating option to reduce potential confusion, which is consistent with previous research on perceived cultural values (e.g., Wan et al., 2007).

We computed the average value dimension scores for both groups separately. Their internal consistency was acceptable (Chinese Canadians: α_opennesstochange_ = 0.80, α_conservation_ = 0.78, α_self-transcendence_ = 0.83, α_self-enhancement_ = 0.75; European Canadians: α_opennesstochange_ = 0.81, α_conservation_ = 0.70, α_self-transcendence_ = 0.82, α_self-enhancement_ = 0.61). Before creating composite scores, we centered each participant’s value rating around his or her mean rating of all 58 values to reduce individual differences in scale use (Schwartz, 2009). Consistent with previous research (Fung et al., 2016), Chinese Canadian participants believed Chinese culture to be higher on conservation (vs. openness to change: *M* = -1.69; compared with 0, *t* = -12.92, *p* < 0.001) and self-enhancement (vs. self-transcendence: *M* = -1.15; compared with 0, *t* = -9.85, *p* < 0.001). Similarly, European Canadian participants also regarded Chinese culture as being higher on conservation (vs. openness to change: *M* = -2.12; compared with 0, *t* = -12.32, *p* < 0.001) and self-enhancement (vs. self-transcendence: *M* = -0.36; compared with 0, *t* = -2.38, *p* = 0.02).

##### Chinese identity

To assess heritage identity for the Chinese Canadian participants, Cameron’s (2004) three-factor social identification scale was used. The scale consists of 12 items and the participants were asked to rate how much they agree with each statement on a 6-point scale from 1 (*strongly disagree*) to 6 (*strongly agree*). The centrality subscale refers to the cognitive importance of Chinese identity and was assessed by four items (e.g., “I often think about the fact that I am a member of my heritage group”). The affect subscale reflects one’s evaluation associated with being Chinese and was also measured by four items (e.g., “Generally, I feel good when I think about myself as a member of my heritage group”). The ingroup ties subscale refers to the sense of connectedness to the Chinese community (e.g., “I have a lot in common with other members of my heritage group”). Internal consistency for the three subscales was satisfactory: 0.74 (centrality), 0.76 (affect), 0.79 (ties). Composite mean scores were thus created for each subscale.

##### Bicultural identity conflict

The 4-item conflict subscale of the Bicultural Identity Orientation Scale (Comănaru et al., 2017) was adapted to assess the extent to which the Chinese Canadian participants experienced tension between Chinese and Canadian cultures. An example item is “There is a conflict within myself between the two cultures I belong to.” The Chinese Canadians indicated their agreement with each item on a 6-point scale (1 = *strongly disagree*, 6 = *strongly agree*; α = 0.77). Responses to the four items were averaged to create composite scores.

## Results

### Data Analytic Procedure

Given the multivariate nature of our outcome variables (i.e., three components of heritage identity), we used path models to examine how FICK affects heritage identification (in both studies). In each path model we tested, we specified the FICK predictor, the mediator(s) of interest, and the identity outcomes. We treated fit of the overall model as evidence that the general effect of FICK on heritage identity was either positive or negative and that the overall effect was mediated. Overall model fit was evaluated with the following fit indices: the confirmatory fit index (CFI), the Tucker-Lewis index (TLI), the root-mean-square error of approximation (RMSEA), and the standardized root-mean-squared residual (SRMR). Standard cutoffs for an acceptable fit are: CFI > 0.95, TLI > 0.95, RMSEA < 0.06, and SRMR < 0.08 (Hu and Bentler, 1999). In keeping with the caution against an uncritical use of simple thresholds for fit indices (Kline, 2011), we considered the chi-square statistic relevant as a statistically significant chi-square could indicate problematic model-data discrepancies in relatively small samples, which was the case in our studies. When the overall model demonstrated a satisfactory fit to the data, we then proceeded to test each indirect or mediated effect, that is, whether each mediator made an independent contribution to explaining the overall effect. All path models were tested with Mplus Version 8 (Muthén and Muthén, 1998/2012).

### Outgroup FICK

We relied on overall profile similarity (Furr, 2010; De Leersnyder et al., 2011) to index FICK. A high correlation between two value profiles indicates similarity in the overall distribution between the values of the two profiles, that is, the two profiles endorse each value relative to the others to the same extent. We created two FICK indices to represent similarity across separate bipolar dimensions: openness to change versus conservation and self-transcendence versus self-enhancement. To calculate FICK openness to change versus conservation (FICK-OC), we created two value profiles. The first value profile was each Chinese Canadian participant’s personal belief across the 22 values representing that dimension. The second was an average value profile of the common Canadian beliefs provided by the European Canadian participants, with each of the 22 values averaged across those participants. FICK-OC was thus calculated as the correlation between the two profiles within each Chinese Canadian participant. Similarly, we used the data corresponding with the self-transcendence versus self-enhancement dimension (22 values) to calculate FICK self-transcendence versus self-enhancement (FICK-SS). A higher score indicates a stronger outgroup FICK in the sense of a stronger belief in Chinese culture being higher on conservation (vs. openness to change) or self-enhancement (vs. self-transcendence). In all the models tested below, Fisher transformations were first performed on the FICK indices.

### Path Model

**FIGURE 2:**
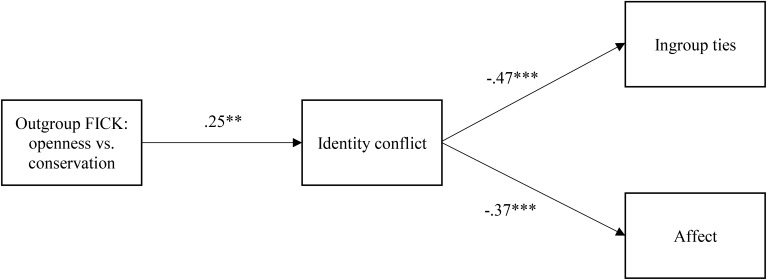
The path model depicting how outgroup FICK in openness to change versus conservation affects Chinese identification via identity conflict (Study 1). FICK, fit in cultural knowledge. Numbers represent standardized regression coefficients. ^∗∗^*p* < 0.01, ^∗∗∗^*p* < 0.001.

As described above, we empirically assessed whether both FICK indices should be included in the path model by examining their correlations with identity conflict, the proposed mediator. Only FICK-CC was correlated with identity conflict, *r* = 0.24, *p* = 0.017. Moreover, because identity conflict was not correlated with centrality (*r* = -0.08), which is consistent with previous findings, we only kept ingroup ties and affect as outcomes (see Table 1). Therefore, the empirically informed path model consisted of FICK-CC (*r*-to-*Z* Fisher transformed), identity conflict, ties, and affect^3^ (see Figure 2).

**Table 1:** Descriptive statistics and intercorrelations of Study 1.

	1	2	3	4	5	6	*M*	*SD*
(1) Outgroup FICK-OC	–						0.57	0.27
(2) Outgroup FICK-SS	0.52***	–					0.46	0.19
(3) Identity conflict	0.25*	-0.07	–				2.20	1.02
(4) Centrality	-0.14	-0.06	-0.08	–			3.70	1.14
(5) Affect	-0.09	0.09	-0.37***	0.30**	–		5.02	0.82
(6) Ingroup ties	-0.22*	-0.04	-0.47***	0.36***	0.42***	–	4.22	1.00

*FICK-OC, fit in cultural knowledge in openness to change vs. conservation. FICK-SS, fit in cultural knowledge in self-transcendence vs. self-enhancement. ^∗^p < 0.05, ^∗∗^p < 0.01, ^∗∗∗^p < 0.001*.

The overall model fit was excellent: χ^2^(2) = 1.84, *p* = 0.40, CFI = 1.00, TLI = 1.01, RMSEA = 0.00, 90% CI [0.00, 0.19], SRMR = 0.029. FICK-CC was shown to have a negative effect on both ingroup ties and affect via identity conflict. We then used the bootstrapping procedure to estimate individual indirect effects (MacKinnon et al., 2007). A significant indirect effect is indicated by a 95% bootstrapped confidence interval that does not include zero. With a bootstrapped sample of 5,000, the indirect effects of FICK on ingroup ties and affect via identity conflict were significant: CI [-0.63, -0.06], CI [-0.09, -0.01], respectively. Therefore, fit between Chinese Canadians’ personal beliefs and common Canadian beliefs in openness to change versus conservation predicted lower ingroup ties and affect through heightened bicultural identity conflict (Figure 2). We also tested the model with FICK-SS as predictor. Although the overall model fit was almost identical [χ^2^(2) = 1.80, *p* = 0.41, CFI = 1.00, TLI = 1.01, RMSEA = 0.00, 90% CI [0.00, 0.19], SRMR = 0.027], FICK-SS failed to predict identity conflict (*p* = 0.50) and as a result, its indirect effects on ingroup ties and affect were not significant: CI [-0.28, 0.52], CI [-0.03, 0.08].

## Discussion

The first study provided initial support for the negative effects of outgroup FICK on heritage identification, specifically for the ingroup ties and affective dimensions of Chinese identity. It also identified one mechanism that underlies this effect: a greater fit between personal beliefs and common Canadian beliefs predicted more bicultural identity conflict, which was in turn associated with a weaker sense of connection with and a less positive evaluation of Chinese culture. This suggests a proximal reason why outgroup FICK negatively affects heritage identification seems to be the experience of bicultural identity conflict. Interestingly, although both Chinese and European Canadians considered Chinese culture higher on conservation (vs. openness to change) and self-enhancement (vs. self-transcendence), it was only their match in the former bipolar dimension that predicted identity conflict. It may be because Chinese Canadians perceived the cultural difference on that dimension to be particularly large or salient. Our path model suggests their belief that openness to change values are much less important and conservation values more important in Chinese culture may make their Chinese identity harder to reconcile with their Canadian identity and that such perceived incompatibility contributes to the experience of identity conflict (Amiot et al., 2007; Huynh et al., 2011).

We also found that identity conflict was not associated with the centrality of Chinese identity, which replicates the null findings found in previous research (e.g., Benet-Martínez and Haritatos, 2005; Cheng et al., 2006). What is common among those null findings is that identification was assessed along the cognitive dimension (i.e., identity importance). Perhaps not surprisingly, this suggests identity conflict is more closely related to affective and ingroup ties dimensions.

## Study 2: Perceived Parental and Outgroup Fick

Study 1 provided initial evidence for the negative effects of outgroup FICK on heritage identification and the role of identity conflict as a proximal explanation for the effects. The goal of Study 2 was threefold: (a) to test both parental and outgroup FICK with different operationalizations from Study 1, (b) to explore another distinct yet complementary mechanism, and (c) to examine whether FICK would facilitate acceptance of stereotype content associated with Chinese Canadians.

We continued to examine outgroup FICK, but unlike Study 1, we elicited perceived outgroup beliefs by asking Chinese Canadians to report their perceptions of outgroup beliefs. Thus, the operational definition of outgroup FICK in Study 2 was the fit between a Chinese Canadian’s personal belief and the beliefs about Chinese values that he or she ascribed to typical Canadians (i.e., perceived Canadian view). Similarly, ingroup FICK was operationalized as the fit between a Chinese Canadian’s personal belief and the belief about Chinese values that he or she ascribed to his or her parents (i.e., perceived parental view). Extending the research that shows parents transmit to children not only their personal values but also values that are perceived to be important in the society (e.g., Tam et al., 2012), we reasoned that parents from immigrant families may also convey values they consider normative to their heritage culture, intentionally or not. That is, Chinese Canadians may form their beliefs about Chinese culture, in part, on the basis of what they think their parents’ view of Chinese culture is. Similar to Study 1, we assumed that matching one’s personal view with perceived parental view implies accepting specific ways of viewing Chinese culture such as higher in conservation but lower in openness to change values. The main question in this study was then to test whether both perceived parental and outgroup FICK would negatively predict heritage identification. It should be noted that given the evidence for the supportive role of parental enculturation practices in facilitating children’s heritage identification (Hughes et al., 2006), perceived parental FICK could similarly predict greater heritage identification if it is assumed to accomplish the same socialization goal. To test that assumption, we correlated parental FICK with measures known to facilitate enculturation. As in Study 1, we calculated parental and outgroup FICK for the two bipolar dimensions separately and relied on exploratory analysis to further test which on which dimension FICK would be a stronger predictor.

To further understand why Chinese Canadians may or may not want to maintain Chinese identity, we turned to broad motivations that energize identity construction. Specifically, Vignoles et al. (2006) found evidence for the robust influence of six motives on identity processes across multiple levels of identity: self-esteem (maintain a positive sense of self), continuity (maintain a sense of continuity across time), distinctiveness (maintain a sense of differentiation from others), belonging (fulfill the need for closeness and to be accepted by others), efficacy (maintain the feelings of competence and control), and meaning (find significance and purpose in one’s life). In general, people are more committed to and happier about identities that satisfy those motives. A broad implication of this work is greater clarity toward understanding why it is that given a realistic range of identity options, people pursue some but avoid others (also see, Vignoles et al., 2008). Thus, we explored whether the negative effects of parental and outgroup FICK could be explained by the frustration of some identity motives.

Finally, we examined another consequence of FICK beyond heritage identification. It stands to reason that if a Chinese Canadian agrees with the beliefs attributed to Canadians about their ingroup Chinese values, they are also likely to accept stereotypes that are targeted specifically at Chinese Canadians. Two fundamental dimensions along which to evaluate social groups are warmth and competence. Based on the stereotype content model (Fiske et al., 2002), Asian Americans are stereotyped as being high on competence (hardworking and successful) yet low on sociability (distant and unfriendly), also known as the model minority stereotype. While the model minority stereotype has been typically studied in the form of hetero-stereotypes (the tendency for Asian Americans to be stereotyped by outgroups, especially European Americans), we were interested in auto-stereotypes (the tendency for Asian Americans to stereotype other Asian Americans; for the distinction between auto- and hetero-stereotypes, see Terracciano and McCrae, 2007; Realo et al., 2009). Extending the model minority research to the Canadian context, we explored whether FICK, particularly outgroup FICK, would predict the willingness of Chinese Canadians to accept the model minority stereotype as characteristic of other Chinese Canadians.

### Method

#### Participants

Participants were 157^4^ self-identified Chinese Canadians (62.4% women) at a central Canadian university, who received partial course credit or volunteered for this study. Their mean age was 19.6 years (*SD* = 2.66). The majority (76.4%) were born in Canada. All but six reported having at least one foreign-born parent.

#### Measures

Participants completed an online survey comprised of the following measures and some demographic questions.

##### Cultural values

Participants rated each of Schwartz’s values in terms of how it characterizes Chinese culture from their own point of view (personal belief), the perspective of their parents (perceived parental view), and that of the mainstream Canadian society (perceived Canadian view). To reduce the cognitive burden on our participants, we administered the Portrait Values Questionnaire (PVQ; Schwartz et al., 2001), which is a widely used alternative to the much longer SVS (e.g., the European Social Survey). The PVQ consisted of 21 items, assessing each of the 10 value types with two or three items. Also different from the SVS is the fact that each item is a short portrait of a person holding a particular value (e.g., He/she looks for adventures and likes to take risks. He/she wants to have an exciting life.). The participants’ task was to rate the extent to which each portrait resembles a typical Chinese person (1 = *does not resemble at all*, 7 = *resembles very well*) from (a) their own perspective, (b) the perspective of their parents, and (c) that of the mainstream Canadian society.

As in Study 1, we created dimensional scores for the three perspectives, respectively, after mean-centering each participant’s responses (personal belief: mean α = 0.70; perceived parental view: mean α = 0.70; perceived outgroup view: mean α = 0.70). The Chinese Canadian participants believed that both their parents and other Canadians believed Chinese culture was higher on conservation (vs. openness to change; *M*_parental_ = -2.08, *M*_Canadian_ = -1.86, both *p*s < 0.001) and self-enhancement (vs. self-transcendence; *M*_parental_ = -1.00, *M*_Canadian_ = -1.08, both *p*s < 0.001). On average, they also personally believed Chinese culture was higher on conservation (vs. openness to change; *M* = -1.58, *p* < 0.001) and self-enhancement (vs. self-transcendence; *M* = -0.91, *p* < 0.001).

##### Chinese identity

We used the same adapted social identification scale (Cameron, 2004) as Study 1, except that the participants responded to a 7-point scale (1 = *strongly disagree*, 7 = *strongly agree*). Internal consistency for the subscales was acceptable: 0.62 (centrality), 0.90 (affect), 0.75 (ties). Composite scores were thus created for each subscale.

##### Identity motives

Consistent with prior work (Vignoles et al., 2006), each identity motive (continuity, meaning, belonging, distinctiveness, efficacy, and self-esteem) was assessed by one face-valid item on a 7-point scale (1 = *not at all*, 7 = *extremely*). Participants were asked to evaluate the extent to which their Chinese identity “gives you a sense of continuity—between past, present, and future—in your life” (continuity), “gives a ‘meaning’ to your life” (meaning),” “make you feel close to other people” (belonging), “distinguishes you from other people” (distinctiveness), “makes you feel effective or competent in doing the things you do” (efficacy), and “give you a sense of self-esteem” (self-esteem).

##### Chinese Canadian auto-stereotypes

To assess Chinese Canadians’ acceptance of heightened competence and deficient sociability as general characteristics of their own group, we adapted the Scale of Anti–Asian American Stereotypes (SAAAS; Lin et al., 2005) in two ways. First, we changed the reference group from Asian Americans to Chinese Canadians for our participants. Second, to tap into auto-stereotypes, we asked the participants to indicate the extent which each statement applied to the Chinese Canadian group on a 6-point scale (1 = *does not apply at all*, 6 = *applies very well*). Sample items are “When it comes to education, Chinese Canadians aim to achieve too much” and “Chinese Canadians do not interact with others smoothly in social situations”. As the αs were satisfactory for both dimensions (competence: 0.81; unsociability: 87), relevant items were averaged to create composite scores. Each subscale was scored such that a higher score represents conformity to the stereotypes (i.e., competence and unsociability).

##### Correlates of parental enculturation practices

We assessed two constructs that have been correlated with parental enculturation practices in previous research: Chinese language proficiency and connectedness to family. On the basis of the finding that parental enculturation practices contributed to children’s heritage ethnic identity partly through increasing heritage language proficiency (Phinney et al., 2001), we asked our participants to self-report their proficiency in understanding, speaking, and reading Chinese on a 5-point scale (1 = *poor*, 5 = *excellent*; α = 0.86). Because parental enculturation practices are also likely to reduce intergenerational conflict and/or promote family cohesion, they were expected to increase children’s connectedness to family (for its association with heritage ethnic identity, see Safdar et al., 2003; for its association with enculturation, see Tao et al., 2017). Connectedness to family was assessed with the 21-item family allocentrism scale (Lay et al., 1998) on a 7-point scale (1 = *strongly disagree*, 7 = *strongly agree*; α = 0.85). Composite scores were created for each scale.

## Results

### Parental and Outgroup FICK

As in Study 1, we operationalized FICK in terms of profile similarity in openness to change versus conservation and self-transcendence versus self-enhancement dimensions separately. Parental FICK-OC and FICK-SS was each calculated as the correlation between a participant’s personal belief and the perceived parental belief across the corresponding values. In a similar vein, outgroup FICK-OC and FICK-SS was each calculated as the within-participant correlation between personal belief and the common belief ascribed to Canadians across the corresponding values. The two set of FICK indices were substantially correlated with each other (see Table 2). Given that (see area “c” in Figure 1), we examined their unique effects in the following path models. Higher parental or outgroup FICK indicates a stronger belief that Chinese culture is higher on conservation (vs. openness to change) or self-enhancement (vs. self-transcendence).

**Table 2:** Descriptive statistics and intercorrelations of Study 2.

	1	2	3	4	5	6	7	8	9	10	11	12	**M**	**SD**
(1) Parental FICK-OC	–												0.59	0.32
(2) Parental FICK-SS	0.54***	–											0.57	0.35
(3) Outgroup FICK-OC	0.64***	0.48***	–										0.51	0.37
(4) Outgroup FICK-SS	0.38***	0.65***	0.58***	–									0.53	0.33
(5) Centrality	-0.10	-0.01	-0.05	0.08	–								4.22	1.24
(6) Ingroup ties	-0.16*	-0.14*	-0.18*	-0.02	0.29***	–							4.52	1.31
(7) Affect	-0.02	0.00	-0.05	0.04	0.26***	0.37***	–						5.74	1.22
(8) Continuity	-0.19*	-0.17*	-0.24**	-0.17*	0.31***	0.38***	0.32***	–					4.07	1.49
(9) Meaning	-0.27**	-0.25**	-0.27**	-0.17*	0.38***	0.44***	0.31***	0.56***	–				3.81	1.78
(10) Belonging	-0.25**	-0.16*	-0.15	-0.07	0.30**	0.63***	0.36***	0.45***	0.53***	–			3.93	1.63
(11) Competence	0.08	0.23**	0.07	0.21**	-0.05	-0.09	-0.12	0.00	-0.06	-0.10	–		4.19	0.81
(12) Unsociability	0.09	0.13	0.15	0.17*	-0.14	-0.25**	-0.33***	-0.19*	-0.16*	-0.25**	0.51***	–	3.3	0.84

*FICK-OC, fit in cultural knowledge in openness to change vs. conservation. FICK-SS, fit in cultural knowledge in self-transcendence vs. self-enhancement. ^∗^p < 0.05, ^∗∗^p < 0.01, ^∗∗∗^p < 0.001*.

### Path Models

As before, we took an empirical approach to building each path model. The first path model involved the three identity components as outcomes and identity motives as mediators, whereas the second path model focused on Chinese Canadian auto-stereotypes (competence and unsociability) as outcomes.

**FIGURE 3:**
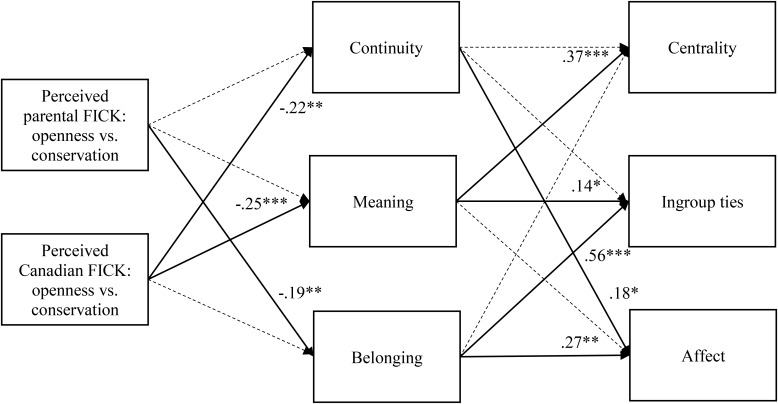
The path model depicting how perceived parental and outgroup FICK in openness to change versus conservation affect Chinese identification via continuity, meaning, and belonging motives (Study 2). FICK, fit in cultural knowledge. Dashed lines represent non-significant paths that were removed from the final model. Solid lines represent statistically significant paths with numbers indicating standardized regression coefficients. ^∗^*p* < 0.05, ^∗∗^*p* < 0.01, ^∗∗∗^*p* < 0.001.

To identify which identity motives should be included in the first path model, we focused on those with significant bivariate correlations with both FICK indices and identity components^5^ (see Table 2 for descriptive statistics). Consistent with Vignoles et al. (2006), all identity components were positively correlated with self-esteem, continuity, belonging, efficacy, and meaning. Parental and outgroup FICK indices were negatively correlated with continuity, meaning, and belonging, but the associations were stronger with parental and outgroup FICK-OC. In light of the bivariate results, we analyzed a path model with continuity, meaning, and belonging as mediators and parental and outgroup FICK-OC as predictors. We included paths between FICK-OC and the three identity motives and paths between those identity motives and all three heritage identity components. Finally, we covaried the identity motives to account for their interrelations. The initial model is shown in Figure 3 (both solid and dashed lines).

The overall model fit was as follows: χ^2^(6) = 3.74, *p* = 0.71, CFI = 1.00, TLI = 1.04, RMSEA = 0.00, 90% CI [0.00, 0.08], SRMR = 0.02. Despite an excellent overall fit, several paths were not significant. In the interest of reducing model complexity, we removed them one by one. Following each removal, the overall fit remained strong and the final fit was as follows: χ^2^(13) = 13.17, *p* = 0.43, CFI = 1.00, TLI = 1.00, RMSEA = 0.01, 90% CI [0.00, 0.08], SRMR = 0.04. Solid lines in Figure 3 represent statistically significant paths. Parental FICK-OC had negative effects on ingroup ties and affect via belonging. Thus, the more Chinese Canadians’ personal beliefs coalesce with the perceived parental view, the less the belonging motive was able to be satisfied, which in turn predicted lower ingroup ties and affect. Outgroup FICK-OC predicted centrality and ingroup ties via meaning. The more Chinese Canadians’ personal beliefs aligned with the perceived Canadian view, the less the meaning motive was able to be fulfilled, which in turn predicted lower centrality and ingroup ties. Outgroup FICK-OC also predicted affect via continuity. The more Chinese Canadians’ personal beliefs aligned with the perceived Canadian view, the less the continuity motive was able to be satisfied, which then predicted lower affect. Bootstrapped confidence intervals based on 5,000 resamples showed that all indirect effects were significant: parental FICK-OC-belonging-ties (CI [-0.45, -0.07]), parental FICK-OC-belonging-affect (CI [-0.41, -0.005]), outgroup FICK-OC-meaning-centrality (CI [-0.35, -0.08]), outgroup FICK-OC-meaning-ties (CI [-0.19, -0.006]), and outgroup FICK-OC-continuity-affect (CI [-0.34, -0.001]).

We also tested the impact of parental and outgroup FICK-SS as predictors. Initial analysis indicated parental FICK-SS had uniquely negative effects on centrality, ingroup ties, and affect via continuity, meaning, and belonging: χ^2^(6) = 6.63, *p* = 0.36, CFI = 1.00, TLI = 0.99, RMSEA = 0.03, 90% CI [0.00, 0.11], SRMR = 0.03. When considered in conjunction with parental and outgroup FICK-OC, however, parental FICK-SS no longer showed unique indirect effects: parental FICK-SS-meaning-centrality (CI [-0.25, 0.002]), parental FICK-SS-belonging-ties (CI [-0.35, 0.24]), parental FICK-SS-continuity-affect (CI [-0.03, 0.004]), and parental FICK-SS-belonging-affect (CI [-0.03, 0.02]). Thus, the effects of parental FICK-SS were subsumed by those of FICK-OC.

**FIGURE 4:**
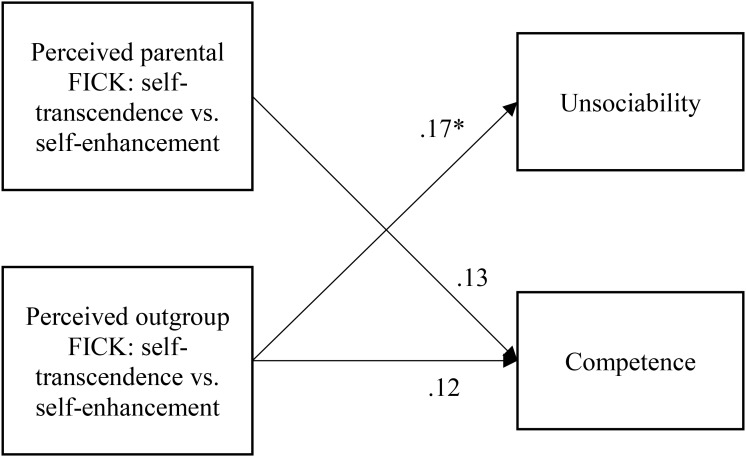
The path model depicting how perceived parental and outgroup FICK in self-transcendence versus self-enhancement affect competence and unsociability auto-stereotypes (Study 2). FICK, fit in cultural knowledge. Numbers represent standardized regression coefficients. ^∗^*p* < 0.05.

For the second path model involving Chinese Canadian auto-stereotypes, we first correlated the four FICK indices and auto-stereotypes (competence and unsociability). Because competence was consistently associated with both parental FICK-SS and outgroup FICK-SS and unsociability was significantly associated with outgroup FICK-SS only (see Table 2), we tested a model with those two FICK indices as predictors (see Figure 4).

The model demonstrated an overall good fit: χ^2^(1) = 0.26, *p* = 0.67, CFI = 1.00, TLI = 1.07, RMSEA = 0.00, 90% CI [0.00, 0.17], SRMR = 0.01. Outgroup FICK-SS predicted unsociability. Neither parental or outgroup FICK-SS, however, uniquely predicted competence. Thus, parental and outgroup FICK in self-transcendence vs. self-enhancement made a collective contribution to the prediction of the competence auto-stereotype, but neither exerted a unique effect.

### Auxiliary Analyses

**FIGURE 5:**
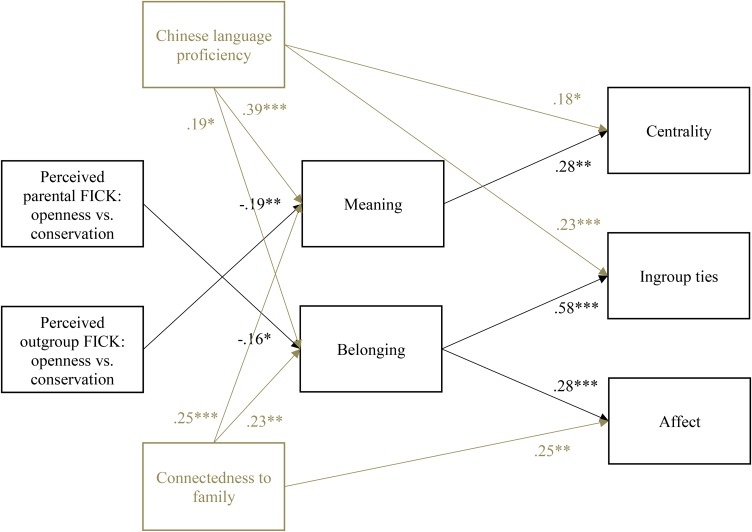
The path model with perceived parental and outgroup FICK in openness to change versus conservation as predictors and meaning and belonging motives as mediators, while Chinese language proficiency and connectedness to family were controlled for (Study 2). χ^2^(14) = 13.47, *p* = 0.49, CFI = 1.00, TLI = 1.00, RMSEA = 0.00, 90% CI [0.00, 0.08], SRMR = 0.03. FICK, fit in cultural knowledge. Numbers represent standardized regression coefficients. ^∗^*p* < 0.05, ^∗∗^*p* < 0.01, ^∗∗∗^*p* < 0.001.

Finally, to test the assumption that parental FICK is similar to parental enculturation practices in functioning to socialize the youth in their heritage culture, we correlated the two parental FICK indices with heritage language proficiency and connectedness to family. Functional similarity would be supported by positive associations. However, all correlations were slightly negative or virtually zero: between FICK-OC and Chinese language proficiency, *r* = -0.11, *p* = 0.19; between FICK-OC and connectedness to family *r* = -0.14, *p* = 0.08; between FICK-SS and Chinese language proficiency, *r* = -0.14, *p* = 0.10; between FICK-SS and connectedness to family *r* = -0.05, *p* = 0.52. We also tested the path model with identity motives as mediators while controlling for Chinese language proficiency and connectedness to family. As displayed in Figure 5, both variables predicted more satisfaction of meaning and belonging motives and higher centrality, ingroup ties, and affect. Notably, parental FICK-OC and outgroup FICK-OC continued to predict lower centrality, ingroup ties, or affect via the thwarting of meaning or belonging needs. The main difference is that compared with Figure 3, only the indirect effects via meaning and belonging were significant: parental FICK-OC-belonging-ties (CI [-0.40, -0.02]), parental FICK-OC-belonging-affect (CI [-0.04, -0.002]), and outgroup FICK-OC-meaning-centrality (CI [-0.24, -0.03]).

## Discussion

We found further evidence for the overall negative effects of outgroup FICK on Chinese heritage identification while extending Study 1 in several ways. First, the stronger the fit between Chinese Canadians’ personal beliefs and the perceived outgroup beliefs, the less the continuity and meaning motives were able to be satisfied. In other words, accepting the perceived outgroup view was less effectual in anchoring young Chinese Canadians’ sense of self-continuity and conferring a sense of significance and purpose. The thwarting of those needs, in turn, predicted lowered heritage centrality, ingroup ties, or affect. Notably, we assessed European Canadians’ perception of how Chinese culture was viewed in the Canadian society in Study 1, whereas Study 2 focused on Chinese Canadians’ perception of how Chinese culture was viewed by other Canadians. Despite this measurement difference, those two views were highly correlated: *r* = 0.94, *p* < 0.001. Importantly, because cultural values were assessed through different instruments across the two studies, we could only correlate the two views at the level of the 10 value types, which likely inflated their similarity. Thus, both studies converge in showing the overall negative effects of outgroup FICK, albeit shown through potentially different mechanisms. What is also consistent is matching on the openness to change vs. conservation bipolar dimension was more psychologically consequential.

Second, the fit between Chinese Canadians’ personal beliefs and the perceived parental beliefs on openness to change vs. conservation was also found to predict lower ingroup ties and affect via the compromised belonging motive. Sharing with one’s parents what Chinese culture values makes it difficult to connect with others in contemporary Canadian society. The fact that accepting the perceived parental view was responsible for some of the negative effects both at the bivariate level and in the path model was not entirely expected. On the one hand, this finding corroborates the pivotal role family plays in enculturation; parents’ view of heritage culture is transmitted, to some extent, to their children, which in turn affects their sense of heritage identity. On the other hand, unlike parental enculturation practices (Hughes et al., 2006), agreement in perceptions of heritage culture had a strikingly negative implication. To find out whether accepting the perceived parental view is similar to parental enculturation practices in functionality, we included two measures known to be affected by the latter: heritage language proficiency and connectedness to family. Neither showed positive correlations with accepting the perceived parental view and consistent with previous research, they uniquely predicted higher heritage identification (Figure 5). Those findings suggest that the tendency for Chinese Canadian young adults to accept the perceived parental view about the ingroup Chinese values lacks the explicit or implicit goal of strengthening the importance of heritage culture and increasing heritage pride.

This study showed another specific consequence of FICK: accepting the stereotypes (i.e., auto-stereotypes) that characterize Chinese Canadians as competent yet lacking sociability. Although it makes sense for perceived outgroup FICK to be the main driving force (Chao et al., 2012), we also found perceived parental FICK to predict competence. The competence auto-stereotype was predicted collectively by parental and outgroup FICK, but neither was strong enough to exert a unique effect. Thus, it seems fair to conclude tentatively that both contributed equally to the competence auto-stereotype, but perceived outgroup FICK uniquely predicted the unsociability auto-stereotype. Another interesting finding is that while the negative effects on heritage identification in both studies were driven by the match on openness to change vs. conservation, the model minority stereotype was more strongly predicted by the match on self-transcendence vs. self-enhancement. This may be because some values represented by the latter bipolar dimension, such as achievement and universalism, are closely connected with what the model minority stereotype connotes: striving for educational and financial success while exhibiting excessive competence perhaps at the expense of the common good (Lin et al., 2005). Finally, the moderate endorsement of competence and unsociability among the Chinese Canadian participants (see Table 2) provides evidence for the existence of the model minority stereotype in Canada (Kil et al., unpublished).

## General Discussion

The present research introduced the notion of FICK and explored its implications for enculturation, particularly identification with heritage culture among Chinese Canadian university students. We conceptualized FICK as the match between Chinese Canadians’ personal beliefs and generalized and close others’ beliefs regarding what values are normative in Chinese culture. In examining others’ beliefs, we included common beliefs about normative Chinese values that are held by or ascribed to Canadians (outgroup FICK, Studies 1 and 2) as well as personal beliefs about normative Chinese values that are ascribed to the parents of Chinese Canadian young adults (parental FICK, Study 2). Overall, the more a Chinese Canadian perceived a similarity between his or her personal beliefs and beliefs ascribed to his or her parents or typical Canadians, the less strongly that person identified with Chinese culture. In Study 1, the negative effects of outgroup FICK could be explained by the fact that it predicted bicultural identity conflict. In Study 2, the negative effects of perceived parental and outgroup FICK were instead mediated by the frustration of three identity motives (continuity, meaning, and belonging). It should be noted that the negative indirect effects of FICK were more consistently manifested in ingroup ties and affect than in centrality (see Figures 2, 3); at the bivariate level, FICK was reliably associated with ingroup ties (see Tables 1, 2). So fit in beliefs about Chinese culture matters more in terms of the feelings of belonging and perhaps personal evaluation. Finally, Chinese Canadians’ tendency to accept the perceived outgroup view predicted their willingness to attribute the unsociability stereotype to other Chinese Canadians, whereas accepting perceived parental and outgroup views combined to predict willingness to attribute the competence stereotype to other Chinese Canadians.

Outgroup FICK in openness vs. conservation values consistently predicted lower heritage identification in both studies, despite somewhat different assessment. Moreover, accepting the perceived outgroup view inclined Chinese Canadians to stereotype their ingroup as lacking sociability and, to a lesser degree, showing excessive competence (Lin et al., 2005). Importantly, the use of values for assessing outgroup beliefs is markedly different from the use of traits in the work on social stereotypes. Values refer to generally positive end-states (even if some values are not endorsed, they are generally not negative), whereas stereotypical traits run the gamut in terms of valence. Thus interestingly, accepting a generally positive depiction of Chinese culture ascribed to the outgroup was predictive of attributing to other Chinese Canadians stereotypical traits that are negative (unsociability) as well as positive (competence). Such reasoning also renders unlikely the explanation that outgroup FICK predicted lower heritage identification because of its overall negative portrayal of Chinese culture.

Perhaps the most surprising finding pertains to the negative effects of parental FICK. At first blush, it contradicts the well-established conclusion, based on research on various ethnic or racial minority families including immigrant Chinese families, that parental enculturation practices foster minority youth’s development of heritage ethnic identity (Hughes et al., 2006; Umaña-Taylor et al., 2006). As the auxiliary analyses of Study 2 showed, however, parental FICK was empirically distinct from Chinese language proficiency and connectedness to family; in the path model that included all of them, they showed opposite effects on heritage identification. As both heritage language proficiency and family connectedness were indicative of enculturation (Phinney et al., 2001; Tao et al., 2017), those preliminary findings suggest that parental FICK in openness vs. conservation values steers young adults away from increased heritage cultural knowledge and pride. For Chinese Canadian young adults at least, perceiving a common ground with their parents with respect to what Chinese culture stands for, particularly on the openness vs. conservation value dimension, not only fails to strengthen personal connections with Chinese culture, but is actually somewhat detrimental.

An important question that arises from this research is, why overall negative effects of both parental and outgroup FICK? As mentioned before, the cultural psychological perspective (e.g., Markus, 2010) distinguishes the outgroup view, understood to be ideas defined or imposed by the outsiders (typically the dominant group), from the ingroup view, which is meanings and values shared within those who identify with the ingroup (e.g., ethnic peers or parents). Among the Chinese Canadian participants, it was found that both outgroup and parental views painted Chinese culture as being higher on conservation (vs. openness to change) and self-enhancement (vs. self-transcendence). Given the striking similarity between the two views, it is sensible that they showed similar effects. The fact that those effects were also negative may thus be attributed to this relatively consensual understanding of Chinese culture. That is, what appears to matter for heritage identity in this case is not so much whether the view reflects an insider or outsider perspective, or whether the view is largely positive or negative, as the content of the view itself. The more a Chinese Canadian person agrees with this understanding of Chinese culture (i.e., higher on conservation but lower on openness values, regardless of where it comes from), the less this person is identified with the culture. Thus, our research suggests that the two sources of ethnic identity (ingroup vs. outgroup) may converge in their effects in some cases, but be contestable in others.

### Implications for Cultural Fit

Is cultural fit always optimal such that the better the fit, the merrier? In a few domains, the answer has been largely yes. For example, there is evidence that people may benefit from emotionally fitting in with their culture (De Leersnyder et al., 2014, 2015). However, some research failed to find support for cultural fit in other domains. A notable exception is the “immigrant paradox” phenomenon, whereby immigrants such as Hispanics who are more acculturated to the United States (e.g., having spent more time or born in the United States) are more likely to report problematic health outcomes (Schwartz et al., 2010; Guendelman et al., 2011). In other words, cultural fit was associated with worse mental and physical health.

The present research contributes to uncovering the circumstances in which cultural fit may be costly. In our approach, we focused on FICK and its intrinsic relation to the maintenance of heritage culture rather than adaptation to the culture of settlement. Our work suggests a main disadvantage of FICK is acquiring a view of heritage culture that does not fully resonate with young adults, which hampers the deepening of individual relationships with that heritage culture. Despite the relatively narrow scope, our results can also speak to when cultural fit is generally good and when it is not. The overall effects of cultural fit may depend on whether the target population is the majority group in a society (Fulmer et al., 2010; De Leersnyder et al., 2014) or minority groups who tend to come from immigrant families and have two or more cultural backgrounds (De Leersnyder et al., 2011; Güngör et al., 2013). In the case of the latter group, cultural fit is not a straightforward phenomenon because it could refer to fit with the majority culture, with the heritage culture, or the switching between the two. Given the multiple ways in which cultural fit can be practiced and the complexity of maintaining different types of fit, it seems reasonable to expect that cultural fit in the general sense may not be uniformly positive (West et al., unpublished). Thus similar to acculturation (Ryder et al., 2000; Schwartz et al., 2010), cultural fit may be better conceptualized as existing along more than one dimension or domain. Our studies further indicate that even when it comes to fit with the heritage culture, what is being matched matters too. We distinguished between personally endorsing the heritage cultural values and matching beliefs about the normative values of the heritage culture. Whereas the former reflects how cultural fit in values is typically defined and is likely to enhance identification (Wan et al., 2007), the latter, dubbed FICK, was found to reduce identification. We maintain that whether cultural fit is adaptive or not depends on its dimension or domain (overall or domain-specific fit with heritage or majority culture) and its content (what is matched).

### Implications for Heritage Identity Maintenance

The overall negative effects of FICK have several implications for the maintenance of heritage ethnic identity among Chinese Canadian young adults. Of particular interest is the role of ingroup FICK in unmooring Chinese Canadians from their heritage. Given the preliminary evidence that ingroup FICK and parental enculturation practices had opposite effects on heritage identification, Chinese Canadian university students may need more than passively shared cultural knowledge in order to truly appreciate their heritage. Considering the need for individuation during young adulthood, our participants may be at a developmental stage where being the recipient of heritage knowledge signals an unreflective rather than a self-initiated approach to learning about their heritage. For Chinese Canadian young adults, the latter may encompass immersion in Chinese culture in somewhat idiosyncratic ways, which may result in less (rather than more) overlapping with their parents’ view of Chinese culture. Motivationally speaking, a self-initiated process implies a more autonomous form of internalizing one’s culture into the self (Chirkov et al., 2003; Downie et al., 2004), which could instead have a more positive influence on heritage identification.

The finding above supports the general observation that enculturation is not devoid of tension or conflict within immigrant families or the larger ethnic community (Farver et al., 2002; Buki et al., 2003; Costigan and Dokis, 2006). In addition to outgroup hassles, minorities also encounter stressors from the family and the larger ingroup (Lay and Nguyen, 1998; Abouguendia and Noels, 2001). The latter may impinge specifically on the maintenance of heritage traditions and the inclusion of heritage culture in the self-identity of minority youth. For instance, Chinese Canadians reported that they were regarded by other Chinese as more Chinese and less Canadian than they felt themselves (Noels et al., 2010). The discrepancy between the self and reflected ingroup appraisals was, in turn, associated with perceived discrimination from other Chinese among those who were Canadian-born (Noels et al., 2010). It is possible that the feelings of discrimination that result from recognizing such an identity discrepancy would propel one to further loosen ties with the Chinese community. In other words, heritage identity maintenance is precarious in that some youth consider their ingroup members or perhaps even their parents pigeonholing their identity claims, which could ironically lead to further distancing from the heritage culture. The present research further highlights the indeterminacy of enculturation by showing that even seeming to have a shared understanding with their parents regarding their heritage culture could backfire.

Earlier, we discussed another reason why both parental and outgroup FICK predicted lower Chinese identification; where Chinese culture is perceived to fall on value dimensions is clearly consequential to identification. Holding an exaggerated or outdated view of Chinese culture that is maximally different from Canadian culture was associated with lower heritage identification. Thus, maintaining a more moderate view of Chinese culture may contribute to heritage identity maintenance. This interpretation suggests that a heritage identity rooted in a more personal or perhaps dynamic view of the heritage culture may be inherently easier to maintain in immigrant contexts, even if such a view diverges from the parental view.

### Limitations and Future Directions

The results of the studies should be interpreted with several limitations in mind. The most apparent is our explicitly exploratory approach. That is, the details of the findings, especially regarding the indirect effects, were not predicted but derived empirically. Given the novelty of this research, however, we think such an approach is justifiable, although it will be important to replicate the overall findings, particularly the negative effects of parental FICK that were somewhat unexpected. As a whole, our findings should be interpreted as tentative and exploratory that are supposed to motivate more confirmatory research. Another limitation is the correlational nature of our data. Although we modeled FICK as a predictor of heritage identification, the underlying causal relations remain ambiguous. It will be fruitful to draw from the literature on heritage identity development among adolescents by employing a cross-lagged panel design. Such a design is a necessary next step as it will demonstrate how the relation between FICK and heritage identification unfolds over time, thus allowing a stronger causal inference.

A measurement-related limitation is that parental and outgroup views in Study 2 were not assessed as actual views, but as views inferred by Chinese Canadians. The first question that results from that measurement choice is the accuracy of perceived views. While we submit views held by parents and other Canadians should not be equated with views that Chinese Canadians estimate they hold (see Footnote 6), we do not think accuracy is a major issue in the present research because it seems reasonable to assume that if heritage identification is to be affected by the match between personal and others’ beliefs at all, what matters is the beliefs of others that are constructed by Chinese Canadians rather than actual beliefs of others (e.g., Su and Costigan, 2009). However, measurement of the perceived parental view may reflect more systematic biases. According to the social projection hypothesis (Krueger, 1996), people have a tendency to project their personal beliefs about the characteristics of a group when estimating what their ingroup members believe about that group. However, projection is less likely when people estimate how outgroup members respond. In other words, Chinese Canadians’ perception of their parents’ views of Chinese culture may be colored by their personal beliefs. A potential consequence is that the match between personal beliefs and the perceived parental view in Study 2 was artificially inflated by the ingroup projection process. Given the evidence for ingroup projection in research on social stereotypes, it will be important to examine whether the match will be reduced when actual beliefs of the parents are assessed and whether the removal of the opportunity to project will produce similar results.

Despite the tentativeness of our findings, we think they can serve as a springboard for theorizing and future empirical investigations. We list a few viable possibilities. One important question is whether the findings regarding Chinese Canadians can be generalized to other minority groups. On the one hand, to the extent that the mechanisms shown to underlie the effects of FICK hold true, the effects are likely to generalize to some extent. For example, if FICK similarly induces other minorities to heighten perceived more differences between their heritage culture and the culture of settlement, that should be associated with lower heritage identification as well. On the other hand, some of the results may be unique to Chinese Canadians. One interesting finding is a moderately strong match between personal beliefs and outgroup beliefs (see Tables 1, 2). It may reflect the use of values that are largely positive in valence to assess FICK or the importance of assimilating the views of generalized others in East Asian cultures (Kim et al., 2010). However, other minority groups may contest or resist outgroup appraisals in anchoring their personal evaluation of the group. A case in point is group differences in collective self-esteem (Crocker et al., 1994). Two components of collective self-esteem are private regard (ingroup evaluation) and public regard (outgroup evaluation). Among African Americans and African immigrants, their private evaluations of the in-group are often dissociated from their perception of generally negative public views. The separation of private from public regard reflects a strategy that protects them from negative outgroup evaluations while helping them maintain a positive group identity on its own terms (Crocker and Major, 1989; Wiley et al., 2008). In contrast, private and public regard tend to be associated positively among Asian Americans and Asian immigrants (Crocker et al., 1994; Wiley et al., 2008). Thus, more stigmatized minority groups may generally demonstrate a lack of a match or perhaps even a mismatch between personal beliefs and outgroup beliefs about their group or between ingroup and outgroup perspectives (Markus, 2010). Compared with Chinese Canadians, a stronger fit with the outgroup view may well affect group identification even more adversely for heavily stigmatized groups.

Another research avenue is to gain a better understanding of what FICK is and its nomological network. First, one specific direction is to build upon the preliminary evidence that parental FICK functions differently from parental enculturation practices for Chinese Canadian young adults. We interpreted the evidence to mean that parental FICK may, in part, indicate an unreflective stance toward their heritage during early adulthood. Even so, parental FICK can be adaptive during adolescence or more so childhood when most enculturation takes place. It remains possible for parental FICK to exhibit a nomological network that indicates family cohesion and heritage exploration during early developmental periods, therefore proving to be beneficial to heritage identity development. Second, related to this point is another limitation of this research. Given the developmental stage of our population, it would have made more sense to focus on fit with the peer belief, which is a more relevant ingroup source to understanding heritage culture. Future research could examine whether peer FICK has positive effects instead. Given what was found, however, we would predict that peer FICK should show positive effects only if the peer view of Chinese culture does not closely resemble the parental or outgroup view.

Third, another unexplored question is whether FICK also affects identification with the majority culture. Future research could examine ways in which FICK increases or decreases identification with the majority culture. For instance, we suggested in Study 1 that in face of identity conflict, one cognitive strategy is to reduce identity complexity by choosing one cultural identity over another (Yampolsky et al., 2016). To the extent that only claiming the dominant identity is a viable option, outgroup FICK could increase identification with the majority culture. If ethnic minorities do not feel they fully belong, outgroup FICK may facilitate further disengagement from the dominant culture instead. Fourth, we brought up the question of whether young adults would benefit from a weaker FICK through a more self-initiated channel of heritage learning. Ferguson et al. (2016) proposed the notion of remote enculturation to denote indirect or intermittent means of heritage learning (e.g., online communication and visiting heritage country). A key difference between remote enculturation and traditional enculturation that is rooted primarily in the role of parents and the local community is that the former is theorized to require proactive action on the part of the young adults, while the latter tends to be initiated by others. For that reason, remote enculturation is supposed to occur later in life, say, after transitioning to university (Ferguson et al., 2016). It thus seems promising to explore whether remote enculturation makes Chinese Canadian young adults rely less on others (ingroup or outgroup members) in understanding their heritage, which may instead contribute positively to heritage identity maintenance. Finally, in conceptualizing FICK, we pointed out that personal beliefs about heritage culture are formed on the basis of ingroup as well as outgroup views. Critically, our studies focused only on FICK, which is the matching of beliefs, but showing a matching effect in and of itself is not the same as showing FICK resulting from ingroup and outgroup influence. A longitudinal or experimental design is needed in order to provide direct evidence for the knowledge sources of individual differences in FICK. More broadly, a better understanding of FICK requires exploring its antecedents as well as its consequences.

Finally, future research could look into the ways in which FICK and cultural fit as commonly defined are similar. A basic tenet of cultural fit is that the more a person is engaged with their sociocultural environment, the more closely attuned his or her psychological tendencies are (De Leersnyder et al., 2011). FICK may be characterized by a similar process such that longer exposure should predict a larger convergence in cultural knowledge. In our studies, Chinese Canadian participants likely differ in their familiarity with outgroup beliefs about Chinese culture, which implies that those with more familiarity should show a stronger fit with outgroup beliefs. We did not measure familiarity or exposure directly, but the use of generational status as a proxy yielded results consistent with the idea. In both studies, Canadian-born Chinese Canadians tended to accept common Canadian beliefs about Chinese culture more than those who were foreign-born^6^. Thus, the former group’s personal beliefs were more strongly influenced by what Canadians generally believe presumably due to their prolonged awareness of those beliefs. The possibility of outgroup FICK increasing with time awaits a systematic investigation.

In closing, for minority youth, there is no fixed heritage culture waiting to be acquired. That is in part because they construct and position their own understanding in relation to ingroup and outgroup source beliefs about their heritage culture. Paradoxically, the overall effects of a closer alignment between their personal beliefs and those source beliefs – expressed as a stronger FICK – are diminished heritage identification for Chinese Canadians. The proximal reasons for the net negative effects are bicultural identity conflict and the thwarting of continuity, meaning, and belonging identity motives. It remains as a challenge for Chinese Canadians during adulthood to carve out a personal understanding of their heritage culture while maintaining a positive orientation toward it.

## Ethics Statement

This study was carried out in accordance with the recommendations of the Research Ethics Office at the University of Alberta (Study 1) and the Office of Research Ethics at York University (Study 2) with written informed consent from all subjects. All subjects gave written informed consent in accordance with the Declaration of Helsinki. The protocol was approved by the Research Ethics Office at the University of Alberta (Study 1) and Office of Research Ethics at York University (Study 2).

## Author Contributions

RZ and KN conceptualized the research. RZ and RL collected the data, which were analyzed by RZ. RZ prepared the manuscript and KN. RL provided critical feedback.

## Conflict of Interest Statement

The authors declare that the research was conducted in the absence of any commercial or financial relationships that could be construed as a potential conflict of interest.

## Footnotes

1 A caveat to note is that most cross-cultural value differences reported in previous research are based on country-level data (Schwartz, 2008), which yielded value dimensions distinct from the two bipolar dimensions derived from individual-level data (Schwartz, 1994). In other words, it can be only inferred from country-level data that Chinese and Canadians cultures differ in dimensions derived from individual-level data (openness to change versus conservation and self-transcendence versus self-enhancement). Recent work, however, indicates a sufficiently high similarity (although not strict isomorphism) between the individual- and country-level value dimensions (e.g., Fischer et al., 2010), thus warranting such an inference.

2 We originally recruited more participants. Their SVS data were first cleaned based on the recommendations of SVS users’ manual (Schwartz, 2009). Nine Chinese Canadian and one European Canadian who used a particular scale anchor 35 times or more were removed from the final samples because their response pattern suggested failure to discriminate among values.

3 Both identity conflict and affect were transformed to reduce substantial skewness and kurtosis.

4 We interspersed four attention checks throughout the survey (Marjanovic et al., 2015). Twelve additional participants were excluded because they failed to answer correctly at least two of those questions.

5 As in Study 1, affect was transformed to reduce substantial skewness and kurtosis.

6 In Study 1, the generational difference was statistically reliable: for openness to change vs. conservation, *t*(100) = 2.85, *p* = 0.005 (Canadian-born: *M* = 0.63, *SD* = 0.26; foreign-born: *M* = 0.51, *SD* = 0.28); for self-transcendence vs. self-enhancement, *t*(100) = 2.20, *p* = 0.03 (Canadian-born: *M* = 0.50, *SD* = 0.18; foreign-born: *M* = 0.42, *SD* = 0.20). However, the difference in Study 2 was not significant despite a consistent trend: for openness to change vs. conservation, *t*(153) = 1.01, *p* = 0.28 (Canadian-born: *M* = 0.54, *SD* = 0.34; foreign-born: *M* = 0.42, *SD* = 0.43); for self-transcendence vs. self-enhancement, *t*(152) = 0.37, *p* = 0.71 (Canadian-born: *M* = 0.54, *SD* = 0.31; foreign-born: *M* = 0.48, *SD* = 0.37). The non-significant result was likely driven by the fact that most Study 2 participants were born in Canada. It should also be noted that there were no consistent generational differences in heritage centrality, ingroup ties, or affect, *p*s > 0.22. Thus, the negative effects of FICK on heritage identification were not due to generational status.
